# Applying Patient and Public Involvement in preclinical research: A co‐created scoping review

**DOI:** 10.1111/hex.13615

**Published:** 2022-10-10

**Authors:** Pádraig Carroll, Adrian Dervan, Anthony Maher, Ciarán McCarthy, Ian Woods, Rachel Kavanagh, Cliff Beirne, Geoff Harte, Dónal O'Flynn, Cian O'Connor, Tara McGuire, Liam M. Leahy, Javier Gutierrez Gonzalez, Martyna Stasiewicz, Jack Maughan, Pedro Jose Gouveia, Paul J. Murphy, John Quinlan, Sarah Casey, Alice Holton, Éimear Smith, Frank Moriarty, Fergal J. O'Brien, Michelle Flood

**Affiliations:** ^1^ School of Pharmacy and Biomolecular Sciences RCSI University of Medicine and Health Sciences Dublin Ireland; ^2^ Tissue Engineering Research Group (TERG), Department of Anatomy and Regenerative Medicine RCSI University of Medicine and Health Sciences Dublin Ireland; ^3^ Science Foundation Ireland (SFI) Advanced Materials and BioEngineering Research (AMBER) Centre Trinity College Dublin (TCD) and RCSI University of Medicine and Health Sciences Dublin Ireland; ^4^ c/o Irish Rugby Football Union (IRFU) Charitable Trust Dublin Ireland; ^5^ Faculty of Sports and Exercise Medicines RCSI University of Medicine and Health Sciences & Royal College of Physicians in Ireland Dublin Ireland; ^6^ RCSI Library RCSI University of Medicine and Health Sciences Dublin Ireland; ^7^ Tallaght University Hospital Dublin Ireland; ^8^ National Rehabilitation Hospital Dún Laoghaire Ireland

**Keywords:** consumer involvement, patient and public engagement, Patient and Public Involvement, public involvement in research, service user

## Abstract

**Background:**

Patient and Public Involvement (PPI) in research aims to improve the quality, relevance and appropriateness of research. PPI has an established role in clinical research where there is evidence of benefit, and where policymakers and funders place continued emphasis on its inclusion. However, for preclinical research, PPI has not yet achieved the same level of integration. As more researchers, including our team, aim to include PPI in preclinical research, the development of an evidence‐based approach is important. Therefore, this scoping review aimed to identify and map studies where PPI has been used in preclinical research and develop principles that can be applied in other projects.

**Methods:**

A scoping review was conducted to search the literature in Medline (PubMed), EMBASE, CINAHL, PsycInfo and Web of Science Core Collection to identify applied examples of preclinical PPI. Two independent reviewers conducted study selection and data extraction separately. Data were extracted relating to PPI in terms of (i) rationale and aims, (ii) approach used, (iii) benefits and challenges, (iv) impact and evaluation and (v) learning opportunities for preclinical PPI. Findings were reviewed collaboratively by PPI contributors and the research team to identify principles that could be applied to other projects.

**Results:**

Nine studies were included in the final review with the majority of included studies reporting PPI to improve the relevance of their research, using approaches such as PPI advisory panels and workshops. Researchers report several benefits and challenges, although evidence of formal evaluation is limited.

**Conclusion:**

Although currently there are few examples of preclinical research studies reporting empirical PPI activity, their findings may support those aiming to use PPI in preclinical research. Through collaborative analysis of the scoping review findings, several principles were developed that may be useful for other preclinical researchers.

**Patient or Public Contribution:**

This study was conducted as part of a broader project aiming to develop an evidence base for preclinical PPI that draws on a 5‐year preclinical research programme focused on the development of advanced biomaterials for spinal cord repair as a case study. A PPI Advisory Panel comprising seriously injured rugby players, clinicians, preclinical researchers and PPI facilitators collaborated as co‐authors on the conceptualization, execution and writing of this review, including refining the findings into the set of principles reported here.

## 1 INTRODUCTION

Actively involving patients and the public in research is increasingly recognized as necessary to ensure outcomes are relevant and beneficial to the people most likely to be affected by them. This is reflected by increasing awareness of the role of Patient and Public Involvement (PPI) in research. PPI is usually defined as research ‘with’, or, ‘by’ members of the public rather than ‘to’, ‘about’ or ‘for’ them.^1^ This definition was originally developed by the advisory group INVOLVE, which has since been integrated into the National Institute for Health Research (NIHR).^2^ More recently, the NIHR definition of PPI has moved away from separating ‘patients’ and the ‘public’, now referring to PPI as public involvement in research.^1^ PPI can involve patients at any stage of a research project, from identifying research opportunities to supporting the dissemination of findings to broader audiences. It can also take many forms, for example, patients and the public participating on steering or advisory committees, reviewing study protocols or collaborating as co‐researchers.^3^ The activities are often mapped to frameworks such as the NIHR research cycle, which describes the stages of research where PPI can be implemented: identifying and prioritizing, commissioning, designing and managing, undertaking, disseminating, implementing and evaluating impact.^1^ Researchers who employ PPI in their studies have reported many benefits such as enhanced research quality and appropriateness, and additional impacts including user‐focused participant information, enhanced recruitment strategies and improved dissemination of findings.^4^

Within clinical research, PPI has become a relatively standard component of research practice. Key stakeholders including funding agencies, regulators and leading journals routinely acknowledge the significant role PPI has to play in improving research and frequently require researchers to provide evidence of PPI in their work.^1^, ^5^ In contrast, the role of PPI in preclinical research is less well established. Preclinical research (meaning basic, fundamental, biomedical, translational or lab‐based research) typically takes place in settings far removed from patients and the public and may seem inaccessible or obscure when compared to clinical research. While some suggest PPI may reduce waste, increase value and improve quality in preclinical research,^6^, ^7^ others caution that it may be more difficult for patients and the public to meaningfully influence research in this setting.^8^, ^9^ This divergence in opinion poses challenges for those exploring the potential for PPI in their preclinical studies. This is further compounded by a limited empirical literature base to guide the selection of applicable PPI approaches or goals.

Our PPI Advisory Panel encountered this challenge when working to develop an evidence‐informed PPI strategy to support a preclinical spinal cord repair project. The project, in the regenerative medicine and tissue‐engineering field, aims to develop an advanced biomaterial‐based ‘scaffold’ platform for spinal cord repair encompassing cutting‐edge science in stem cell and gene therapy. The project is funded through a research partnership between the Royal College of Surgeons in Ireland University of Medicine and Health Sciences (RCSI), the Irish Rugby Football Union (IRFU) Charitable Trust and the Science Foundation Ireland (SFI) Advanced Materials and Bioengineering Research (AMBER). The PPI Advisory panel was established in 2019 and comprises three seriously injured rugby players, three clinicians, as well as several preclinical researchers and facilitators. The panel meets biannually to oversee and advise on the preclinical research progress and collaborate on PPI initiatives. At an early meeting, the team identified that a strategy would be useful to support the PPI activity and that this should be informed by existing literature where possible.

The team was aware of two existing review studies at that time which focused narrowly on specific areas of antimicrobial drug development and genomics,^10^, ^11^ but none that mapped empirical preclinical PPI literature across disciplines. Panel members agreed that a scoping review would be useful, and collaboratively developed a protocol for the study.^12^ In the intervening period, another scoping review was published that explored patient engagement in preclinical laboratory research. However, this study had a more general aim and broader definition, including engagement and involvement activities, and included secondary as well as primary literature.^13^

The research question for this scoping review study was how do researchers incorporate PPI in preclinical research?^12^ The aims of this review were to identify and map the current empirical literature on PPI in preclinical research to identify why researchers used PPI, the volume and range of approaches used, the benefits and challenges encountered, the impacts they reported and potential applications for our own PPI strategy. We planned to synthesize the initial findings collectively as a group comprised of PPI Advisory Panel members to ensure that the findings reflected the perspectives of the entire team. We aimed to use the review findings to inform the development of a PPI strategy tailored for a preclinical spinal cord repair project.

## 2 METHODS

### 2.1 Guiding framework

This review was conducted according to the Joanna Briggs Institute (JBI) guidance for conducting scoping reviews.^14^ This framework builds on the methodology outlined by Arksey and O'Malley^15^ and Levac et al.^16^ and provided the structure for identifying eligibility criteria, refining search strategy, selecting sources of evidence, extracting data, analysing evidence, presenting results and consulting with stakeholders. The review is reported with reference to these JBI guidelines and the Preferred Reporting Items for Systematic Reviews Extension for Scoping Reviews (PRISMA‐ScR; Supporting Information: Appendix 1).^17^

### 2.2 Eligibility criteria

Studies were considered eligible for inclusion if they were primary empirical studies involving PPI in preclinical research settings and involved interacting with patients and/or the public directly. Only studies written in English were included due to translation costs and the risk of misinterpretation. No limitations were placed on the study location or date of publication. ‘Patient engagement’^18^ and ‘community‐based participatory research’ (CBPR)^19^ approaches overlap with PPI to a certain degree as they involve patients. However, there are differences relating to the definition, approach, origins and the level of ownership held by researchers and therefore, these were not considered eligible for inclusion.^18^, ^20^ Studies describing contact with representative organizations with no direct involvement of patients and/or the public were excluded. While the majority of studies would likely be clearly clinical or preclinical, it was possible that there could be some degree of overlap. In cases where the nature of the research was not immediately clear, the following question was considered: ‘Does this research have an immediate clinical application?’ If an immediate clinical application was identified, the study was excluded.

### 2.3 Information sources and search strategy

The search strategy was developed in consultation with a specialist librarian PM who is a co‐author of this review. Search terms were determined with input from advisory panel members. Recognizing the variation in terminology used to describe PPI internationally, terms relating to patient engagement and CBPR were included in the search strategy. This ensured that studies including PPI but using different terminology were captured in the search. However, they were excluded at the study selection stage if their approach was not considered to be PPI. To identify preclinical research studies, databases were searched for specific preclinical research techniques. Data were sourced using an academic database search, and manually searching citation lists for included studies The following databases were searched: MEDLINE (PubMed), PsycInfo, EMBASE and Web of Science Core Collection. The search strategy used in EMBASE is provided in Table 1. Databases were searched from their inception to May 2021. An updated search was run including additional search terms not covered by the original search strategy in August 2021.

**Table 1:** EMBASE search strategy

EMBASE
A
‘patient participation’/exp OR (patient NEXT/1 participation) OR (patient NEXT/1 participants) OR (participatory NEXT/1 research)
B
‘biomedicine’/exp OR (biomedical NEXT/1 science*)
‘translational medicine’/exp OR (translational NEXT/1 medicine)
‘medical research’/exp OR (biomedical NEXT/1 research)
‘animal experiment’/exp OR (animal NEXT/1 experimentation)
(animal NEXT/1 models)
‘bioassay’/exp OR (biological NEXT/1 assay*)
‘drug development’/exp OR (drug NEXT/1 development)
(immunologic NEXT/1 techniques)
‘cytology’/exp OR (cytological NEXT/1 techniques)
‘device approval’/exp OR (device NEXT/1 approval)
‘chemical analysis’/exp OR (chemistry NEXT/1 techniques)
‘in vitro study’/exp OR (in NEXT/2 vitro NEXT/2 techniques)
/OR
C
A AND B
D
Limited to Embase and Medline records, excluding Medline only
Rerun searches 12/05 (*n* = 9138) limited to 2020/2021 excluding Medline only

### 2.4 Article selection

**Figure 1:**
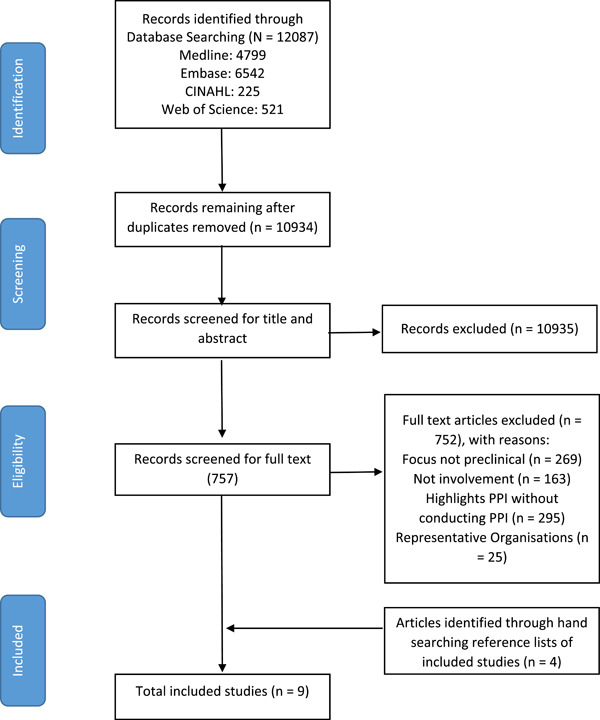
PRISMA flow diagram

The article selection process is outlined in the PRISMA‐flow diagram (Figure 1). Records identified in the database searches were imported into EndNote and duplicates were removed by P. C. Two reviewers P. C. and M. F. conducted screening and study selection according to JBI guidelines for conducting scoping reviews.^14^ In advance of commencing study selection, each reviewer independently screened the titles and abstracts of five articles before comparing the application of eligibility criteria to ensure consistency. Following this, both researchers used Endnote to screen the title and abstract of each record independently before meeting to compare results and ensure consistent application of the eligibility criteria. Discrepancies of opinion regarding studies deemed potentially eligible were resolved through discussion between P. C. and M. F. If consensus could not be reached, a third reviewer F. M. made the final decision. For full‐text screening, both reviewers again completed an initial review of five studies before meeting to compare results. Both then independently reviewed the remaining full texts for eligibility, and discrepancies were resolved through discussion or consultation with the third reviewer.

### 2.5 Data extraction

Two reviewers P. C. and M. F. conducted data extraction using a data extraction form developed in accordance with the JBI guidelines^14^ and the study protocol.^12^ The following information was extracted for each included study:

(1) author(s);
(2) year;
(3) title;
(4) country of origin;
(5) the scientific discipline/context of the study;
(6) who took part in the PPI component (PPI Contributors);
(7) the reason for conducting PPI (PPI Rationale);
(8) what researchers sought to accomplish with PPI (Aims of PPI);
(9) which PPI approach was used (PPI Format);
(10) how PPI was implemented (PPI Methods);
(11) stages of implementation according to NIHR^1^;
(12) benefits associated with PPI;
(13) challenges associated with PPI;
(14) impact of PPI on study;
(15) if/how PPI was evaluated;
(16) elements with potential application within our preclinical PPI project.

### 2.6 Narrative synthesis

One reviewer P. C. conducted a narrative synthesis of the findings according to the guidelines set out by Popay et al.^21^ No quality assessment was conducted.

### 2.7 PPI

Three members of the PPI Advisory Panel are PPI contributors and were involved in the project from the beginning, including project planning and protocol development stages.^12^ Completed data extraction forms were circulated to PPI Advisory Panel members in advance of a scheduled meeting. The extracted data were discussed in terms of themes/trends identified by the patient partners and other panel members, aspects relevant to the spinal cord repair project, and aspects relevant to preclinical PPI more generally. Perspectives were captured using a digital whiteboard, synthesized afterwards by P. C. and M. F., and incorporated into the discussion.

## 3 RESULTS

### 3.1 Study characteristics

A total of 12,087 studies were identified through database searching. Duplicates were removed and studies underwent title and abstract screening to eliminate studies that did not meet the inclusion criteria. After this process, 54 studies remained for full‐text review. After the full‐text screening, nine studies were included in the final review (Figure 1). Key characteristics of the included studies are summarized in Tables 2, 3, 4. Five studies were from the United Kingdom,^22^, ^23^, ^24^, ^25^, ^26^ two from the Netherlands,^27^, ^30^ one from Belgium^28^ and one from the United States of America.^29^ Eight studies were published between 2010 and 2020,^22^, ^24^, ^25^, ^26^, ^27^, ^28^, ^29^, ^30^ with the remaining study published in 2008.^23^ Two studies were in the context of medical device development,^23^, ^27^ one in drug development,^29^ one in translational rheumatology research,^22^ one in asthma research,^26^ one in vaccine development,^28^ one in microbiology research,^24^ one in autism research^25^ and one in psychiatric genomics research.^30^

**Table 2:** Characteristics, rationale and aims of included studies

References	Title	Country	Discipline/context	PPI contributors (who were involved)	Rationale for conducting PPI	Aims of PPI component
Birch et al.^22^	Development and formative evaluation of patient research partner involvement in a multidisciplinary European translational research project	The UK and Europe	Translational rheumatology	People with rheumatoid arthritis and people with an elevated risk of developing rheumatoid arthritis.	To ensure the quality and relevance of research, and to facilitate effective translation and dissemination of the research findings.	To capture individuals' thoughts about being at risk of developing rheumatoid arthritis, their acceptance of predictive testing and preferences for risk communication. A further aim was the development and evaluation of informational materials for those at risk of developing rheumatoid arthritis.
Bridgelal Ram et al.^23^	Issues and challenges of involving users in medical device development	UK	Medical product development	Adults with epidermolysis bullosa (EB), their carers; and specialist nurses in the management of complex wounds. Along with the EB nurses, the multidisciplinary team comprised designers, materials scientists, engineers and a pharmacist.	Previous EB products failed to manage adequately the problems experienced by those with EB concerning both materials used and product design. PPI aimed to involve product users in the design of new products in an attempt to address these issues.	To elicit data on current medical products, and identify new areas for research.
Grier et al.^24^	Finding and engaging patients and the public to work collaboratively on an acute infection microbiology research public panel	UK	Microbiology	People with experience of being hospitalized with a serious infection (or someone very close to them).	Patient input may inform the recruitment strategy of a large and diverse PPI panel by identifying potential avenues for recruitment.	To recruit participants for a PPI Advisory Panel.
Russel et al.^25^	Selective Patient and Public Involvement: The promise and perils of pharmaceutical intervention for autism	UK	Biomedical autism research	People with autism, friends, family and people who worked with children or adults with autism.	To establish what is acceptable and not acceptable to various members of the autism community regarding drug development.	To assess the diversity of community views to illustrate how PPI was used in the biomedical context.
Supple et al.^26^	From tokenism to meaningful engagement: best practices in patient involvement in an EU project	UK	Asthma research	Patient representatives and representatives from patient organizations.	To establish good practice for information and evidence on patient involvement.	To provide the patients' perspective, support, and insight into the project's research and dissemination processes; helping the research ‘stay connected’ to the patients' experience and needs.
Elberse et al.^27^	Patient involvement in a scientific advisory process: Setting the research agenda for medical products	The Netherlands	Medical product development	Patients, ‘Expert’ Patients, informal carers and nonpatient representatives across 15 disease domains.	To establish a research agenda responsive to the needs of ‘end users’ regarding future medical products and to make efficient use of limited research resources.	To provide input from patient groups of 15 disease domains to the national health advisory committee, which they could use in their advisory process to establish advice on a national research agenda for medical products.
Baart and Abma^30^	Patient participation in fundamental psychiatric genomics research: a Dutch case study	The Netherlands	Psychiatric genomics	Members of patient groups (Schizophrenia), patient group board members, research assistants and social scientists.	Funders mandate to improve the researcher's interactions with patients to secure more funding.	To bring together and integrate various knowledge sources, scientific and experiential.
Sohy et al.^28^	Outside in–inside out. Creating focus on the patient—a vaccine company perspective	Belgium	Vaccine development	Employees at a vaccine production company.	Encouraging vaccine producers that they are vaccine ‘patients' so that they can better provide input into the vaccine production process as patients rather than solely as producers.	To promote the understanding that employees in a vaccine company are themselves ‘vaccine patients’, and to create a bridge between employees' day‐to‐day activities and their impact on patients.
Chalasani et al.^29^	Enhancing the incorporation of the patient's voice in drug development and evaluation	USA	Drug development	Patients, caregivers, patient advocates and advocacy groups, healthcare providers, professional societies, scientific and academic experts, drug developers and others.	Funding body requirements to involve patients.	To engage patients and elicit their perspectives on two topics: (1) the most significant symptoms of their condition and the impact of the condition on daily life and, (2) their current treatment approaches.

Abbreviation: PPI, Patient and Public Involvement.

**Table 3:** Application, benefits and challenges of included studies

References	Format used	PPI methods	NIHR stages of implementation	Benefits	Challenges
Birch et al.^22^	Patient Research Partners (PRPs)	PRPs were recruited via links with several patient groups and they collaborated with researchers throughout a large‐scale preclinical research project on a number of research activities. This included contributing to research materials, assisting with funding applications and collaborating on dissemination strategies. PPI Training was provided on an ad hoc basis for PRPs.	Identifying and prioritizing, designing and managing, undertaking, disseminating, evaluating impact	Researchers and PRPs both reported the value of building relationships with each other, both noting they developed their knowledge and communication skills. This facilitated the effective dissemination of research findings. By building relationships with PRPs, researchers developed a series of unexpected spin‐off projects such as creating patient‐informed educational resources to communicate scientific concepts to people living with rheumatoid arthritis.	The study reported that despite including an evaluation study, its findings are unlikely to be representative of the patient population due to most PRPs being active members of patient organizations and therefore were already fully engaged with the idea of becoming involved in research.
Bridgelal Ram et al.^23^	Workshops	Informed by the Knowledge Transfer model. Involved informal workshops to carry out brainstorming sessions. Population recruited through EB nurses and meetings held in conjunction with clinics. Initial meetings were to assess current wound care products and provide a knowledge base for future products. Approach gathered data and formed new ideas for product design driven by user needs. Brainstorming workshops took place afterwards with recruitment led by EB nurses, as they knew their patients best. The workshop format allowed the gathering of rich data from varying perspectives, allowing participants to voice their ideas and reflect on the responses of others. As previously mentioned, it also resulted in invitations to patients' homes to observe dressing changes and take photographs where appropriate. Their concepts and feedback were brought to a design team to translate into products.	Identifying and prioritizing	The study reported that initial collaboration between patients and researchers led to invitations from patients to unexpected yet impactful opportunities such as visits to patients' homes to see how they manage their condition.	Many workshops were rescheduled due to participants being unwell. Flexibility was required in planning, postponing and rescheduling workshops due to PPI contributors becoming unwell meaning they could not attend.
Grier et al.^24^	One 2‐h meeting to provide information about the scientific research and inform a PPI panel recruitment strategy	Participants were invited to a meeting where they could meet the research team and ask questions about the PPI panel without committing. A role profile was developed so people could see what was expected of them and what they could expect from the research team.	Identifying and prioritizing	Hosting a public event for prospective panel members to attend meant that researchers could clarify what PPI meant and what they could expect from being part of a PPI Advisory Panel. This ensured potential PPI panel members understood what it meant to be involved before committing. Meeting attendees were also asked to fill in an expression of interest form, allowing researchers to recruit a more diverse PPI panel in terms of age, experience and background.	Researchers describe the process of recruiting a PPI panel as time‐consuming and noted a lack of success early in the study. The study also notes that PPI has costs for time and funding, with significant time spent seeking PPI training for researchers. Finally, researchers noted that despite recruiting a diverse PPI Advisory Panel, the panel may not be fully representative of people with experience of serious infliction.
Russel et al.^25^	Public meeting/email feedback	A promotional video was shown at a public event regarding a research agenda for autism. Attendees were members of the autism community and could submit their feedback on the video at the event or via email so that the research team could understand how people viewed the suitability of their research agenda.	Designing and managing	None identified.	The study notes that if PPI is required by funding bodies there needs to be further training on conducting PPI correctly. Researchers should note that their PPI contributors' viewpoints may not be representative of an entire patient population.
Supple et al.^26^	PPI Advisory Group	A Patient Input Platform (PIP) was established at the start of the project so researchers and PPI contributors could work together to provide the patients' perspective, support and insight into the project's different research and dissemination processes, with a view to helping the research stay ‘connected’ to the patient experience and needs.	Identifying and prioritizing, designing and managing, disseminating	Researchers reported that PPI contributors were a source of motivation for them when faced with difficulties in the research project.	The English language was a barrier to adequate representation.
					Limited travel capacity for some PPI contributors living with serious conditions. Duration of commitment over long‐term projects.
					PPI contributors did not possess the scientific knowledge to have meaningful discussions around aspects of research such as specific research techniques.
					Difficulty persuading preclinical researchers to embrace PPI.
Elberse et al.^27^	Interviews	A participatory approach based on the dialogue model was used comprising four phases: (1) exploration, (2) consultation and prioritization, (3) integration and (4) follow‐up.	Identifying and prioritizing	The study described that PPI contributors felt empowered from taking part in the PPI process. Becoming involved in research, sharing knowledge and seeing a tangible impact resulted in PPI contributors valuing their experiential knowledge more. This led to several of the PPI contributors developing their own research agendas from the patient perspective in collaboration with patient organizations.	The study reported concerns about the representativeness of PPI contributors.
					The authors suggested that PPI contributors may have been indirectly influenced by pharmaceutical companies due to their ongoing use of medical products, although no explicit evidence of this was apparent. Researchers also suggested that it was difficult to conduct PPI in a way that was narrow enough to meet the needs of their research (articulating needs for medical products) while also being sufficiently broad for patients to contribute based on their own experience.
		In phase 1, patients were identified through existing patient organizations. 29 semistructured exploratory interviews were conducted to pilot the appropriateness of research methods with potential participants.			
		Phase 2 comprised semistructured in‐depth interviews and focus groups with patients, expert patients, carers and nonpatient representatives to gather perspectives relating to product development. For focus groups, an exercise was used where participants started by discussing their personal experiences to assist patients in articulating needs for medical products and looked at the argument underlying that need.			
		During Phase 3, patient input was analysed, taking into account similarities, relations and differences between patient groups. The report was written up.			
		In Phase 4, follow‐up interviews were held with patients taking part in the study to gain insight into the usefulness of PPI.			
Baart and Abma^30^	PPI Advisory Panel and interviews	Interviews and focus groups were held to ask patients and scientists what they wanted to know about schizophrenia. This led to identifying areas of common interest between patients and scientists. Once common ground was established, patients made practical recommendations for improved dialogue between scientists and the public and collaborated on a series of activities to improve the interaction between researchers and patients such as a jointly delivered conference workshop.	Identifying and prioritizing, disseminating	Researchers found that engaging in the PPI process helped identify common ground between researchers and patients. This helped form a relationship between researchers and patients, which was used as a basis for interaction with patients—for exchanging information and discussion.	The study reported that the dialogue model, used as a framework for PPI, was a good starting point for interaction between patients and researchers but not necessarily suited for preclinical research. This is due to the long‐term nature of the research, concerns around a lack of subjectivity by PPI contributors being incompatible with preclinical research, and representativeness of viewpoints from PPI contributors of the general patient population. The study also noted an initial scepticism of preclinical researchers towards PPI.
Sohy et al.^28^	Interviews and PPI champions	In face‐to‐face interviews, 40 members of staff were invited to identify barriers and enablers to patient focus in their work. Researchers used these themes to create a framework for PPI activities aiming to increase employees' contact with patients in daily work. Activities included hosting webinars with patients, obtaining patient insight on relevant topics, engaging with patient organizations and creating Voice of the Patient (VoP) Champions who promote PPI within the workplace.	Identifying and prioritizing, undertaking, disseminating, evaluating impact	During a PPI focus group discussion, employees at the vaccine production company came up with their own initiative: nominating a “designated patient” at company meetings. The designated patient would represent the patients' interests in the matter being discussed at meetings.	The strength of the evaluation study is limited by the lack of baseline measures before taking the evaluation survey. This study also noted that PPI contributors were employees of the company producing the vaccines in question.
Chalasani et al.^29^	Panel meetings	Panel meetings comprised of patients, caregivers and representatives—formed for a public meeting. Meetings start with panel members sharing their experiences of living with a particular condition, followed by a semistructured, large group facilitated discussion that encourages participation from other patients, caregivers and patient representatives attending in‐person and via webcast. Patient input gathered from meetings identified areas of unmet need and new outcome measures for clinical trials.	Identifying and prioritizing	Patient input gathered from panel meetings primarily informed drug development programmes. However, this input also had wider use in identifying areas of unmet need in patient populations, developing new outcome measures for clinical trials, planning follow‐up workshops and identifying patient representatives to serve on advisory committees.	Panel meetings were limited to focus on a single disease at a time, meaning many other disease areas could not be addressed by this format. Researchers also had limited meeting space and staff resources to host meetings.

Abbreviations: NIHR, National Institute for Health Research; PPI, Patient and Public Involvement.

### 3.2 Rationale and aims for including PPI

The authors of the included studies discussed several rationales and aim for including PPI (Table 2). In four studies, researchers reported using PPI to improve the quality and relevance of their research by achieving various aims.^22^, ^23^, ^26^, ^27^ For example, PPI was included to make efficient use of funding resources,^27^ identify areas of unmet need,^23^ and facilitate dialogue between patients and researchers.^22^, ^26^ Similar aims and rationale are reported by Sohy et al.,^28^ who described incorporating PPI to create a bridge between employees at a pharmaceutical company producing vaccines and the impact of their vaccines on recipients.

Two studies employed PPI to meet funding body requirements.^29^, ^30^ In one study, preclinical researchers were directed by their main funding body to improve their level of interaction with patients as a prerequisite for further funding.^30^ Initially, researchers described the idea that patients could be involved in preclinical research as ‘impossible’ and ‘new‐fangled nonsense’. However, they responded to this requirement by implementing a PPI programme that aimed to facilitate dialogue between scientific researchers and patients; increasing the knowledge base of the research team. Directives from national funding bodies also provided the rationale for another study, which aimed to obtain patients' perspectives on specific diseases and their currently available treatments.^29^ In this study, researchers conducted PPI to ensure that patients' voices were represented in drug development research. The PPI component aimed to provide patients with an opportunity to discuss their condition with researchers under two topic areas: (1) the impact of the most significant symptoms on their daily life and (2) their current treatment approaches.

Researchers also reported incorporating PPI to receive public input for guiding specific research projects/project outcomes. In one study, researchers conducted PPI aiming to obtain feedback on the appropriateness of a pharmaceutical research agenda for autism.^25^ This was done to ensure that those potentially impacted by the outcomes of their research would have their voices represented, and would be provided with the opportunity to comment on its suitability. In another study, preclinical researchers met with patients to discuss the recruitment of PPI panel members. Researchers reported that it is more difficult to recruit PPI panel members for a preclinical research study. Therefore, meeting with patients and receiving input on the recruitment process helped design a more participant‐friendly recruitment strategy for a PPI panel.^24^

### 3.3 Volume and range of PPI approaches

Preclinical researchers employed a variety of formats when conducting PPI (Table 3). Included formats were described as panel meetings,^29^ workshops,^23^ PPI advisory groups,^26^, ^30^ interviews,^27^, ^28^, ^30^ Patient Research Partners (PRPs),^22^ PPI champions,^28^ public meetings^24^, ^25^ and gathering input via email.^25^ Preclinical researchers also described PPI implementation at a variety of stages of the research cycle, including identifying and prioritizing,^22^, ^23^, ^24^, ^27^, ^28^, ^29^, ^30^ designing and managing,^22^, ^25^ undertaking,^22^, ^28^ disseminating^22^, ^26^, ^28^, ^30^ and evaluating impact.^22^, ^28^ When extracting the stages of implementation of included studies, it became evident that no studies conducted PPI at the commissioning or implementing stage of the NIHR research cycle.^1^

Panel meetings were described by researchers as structured meetings of patients, caregivers and patient representatives to engage in dialogue around specific conditions.^29^ Similarly, workshops were described as informal meetings of researchers, patients and their carers to discuss current treatment options relating to their condition and collaborate on potential solutions.^23^ Patient input from these workshops was then provided to a design team to translate into design concepts, novel technologies and new products.^23^ Another PPI approach used was PRPs. PRPs describe embedded researchers who collaborate with the scientific team throughout the research project on several research activities.^22^ These activities include attending scientific meetings, contributing to and co‐authoring research papers and informing disseminated research materials.^22^ A similar approach was reported in Sohy et al.,^28^ describing the involvement of ‘PPI champions’ in their research. In this approach, a PPI contributor is integrated into the research team as a patient representative. PPI champions act as the voice of the patient and promote their interests throughout the research cycle.^28^

Three studies used interviews to conduct PPI.^27^, ^28^, ^30^ In one study, preclinical researchers and patients were asked what they would like to know regarding their condition/area of research (schizophrenia). In answering these questions, areas of common interest were identified between researchers and patients such as condition aetiology.^30^ Once a shared understanding was established, patients made practical recommendations for improved dialogue between scientists and patients, and both participated in a series of activities to help facilitate interaction, such as jointly hosted workshops by researchers and PPI contributors. Another study employed interviews to gain patient perspectives regarding medical product development.^27^ This involved 23 semistructured in‐depth consultation interviews with patients, patient carers and patient representatives. Interviews were then supplemented by 15 focus groups. In these interviews and focus groups, patients articulated their needs, which helped to inform the development of a research agenda.

Another approach used by preclinical researchers seeking to incorporate PPI was PPI Advisory Panels.^26^, ^30^ One study on identifying biomarkers in respiratory disease used a PPI Advisory Panel, describing their approach as a ‘Patient Input Platform’.^26^ In this approach, patient organizations were invited to provide input into a grant proposal for the research project. Patients from these organizations were then included on multiple boards overseeing the work of the project, provided feedback on project progress and acted as collaborators with researchers on project design and dissemination strategies.^26^

Researchers also conducted PPI by hosting meetings to gather input from patients. In these meetings, patients were invited to attend and provide feedback to researchers regarding their research agenda. In one study, researchers aiming to recruit a PPI panel held an open event for prospective PPI panel members to meet the research team and ask questions about the role of the panel members without committing to join.^24^ In another study, a public meeting was held for people with autism, their friends and family, and people who worked with children or adults with autism. Researchers presented a promotional video describing a pharmaceutical research agenda for autism, and attendees were invited to submit feedback at the meeting or via email, to voice their opinions regarding its suitability.^25^

### 3.4 Benefits and challenges

The incorporation of PPI resulted in many benefits for preclinical researchers (Table 3). In one study, preclinical researchers reported improved communication skills after building relationships with PPI contributors throughout their research project.^22^ Moreover, building such relationships resulted in further research opportunities which may not have come about without PPI. For example, in one study, researchers conducting PPI were invited to visit patients' homes to observe how they manage their conditions.^23^ Other researchers reported that by conducting PPI with employees in a vaccine production company, staff saw opportunities for enhanced engagement such as nominating an employee to represent patient interests at company meetings.^28^

PPI also benefited studies by connecting preclinical researchers with the needs of patients, potentially strengthening understanding and improving research relevance. One study reported that PPI identified areas of common ground between researchers and PPI contributors, which served as a basis for discussion and exchanging knowledge.^30^ Empowerment and motivation were also benefits of conducting PPI. One study reported PPI contributors feeling empowered from contributing to the PPI process.^27^ Becoming involved in research, sharing their experiences and seeing a tangible impact from their input resulted in PPI contributors identifying value from their experiences.^27^ Similarly, researchers were motivated by their interactions with PPI contributors when faced with difficulties in their scientific research.^26^

Studies also reported several challenges. One challenge was that PPI contributors might not represent the diverse set of views held by patients. Seven studies reported this challenge.^22^, ^24^, ^25^, ^26^, ^27^, ^28^, ^30^ PPI contributors were typically self‐selected to become involved in research, meaning they may not represent the viewpoints of a typical patient or member of the public but rather reflect the views of one already interested/involved in a particular aspect of the research programme.^22^, ^24^ One study refers to this as ‘selective PPI’ where only a limited/sympathetic viewpoint is included.^25^ Similarly, researchers who included an evaluation study reported the representativeness of the PPI contributors was a limitation of their work.^22^, ^28^ Researchers also considered PPI as time and resource‐intensive.^24^, ^29^

Another barrier to preclinical PPI was a lack of training and awareness of PPI methodologies.^24^, ^25^ Two studies reported difficulty in persuading researchers of the merits of PPI for preclinical research due to concerns around the ability of PPI contributors to provide a tangible impact on scientific research.^26^, ^30^ Other challenges for preclinical PPI included contributors becoming unwell,^23^ and the suitability of PPI for preclinical research projects which progress slowly and require a long commitment from contributors.^26^, ^30^

### 3.5 Impact and evaluation

A number of impacts were reported in the studies included (Table 4). In one study, researchers reported that PPI directly influenced their funding application and recruitment strategy for participants to provide biological samples for testing by preclinical researchers.^26^ Another impact of PPI on preclinical research was by shaping research agendas, with five studies reporting this as an impact.^23^, ^25^, ^27^, ^29^, ^30^ For example, two of these studies used PPI to articulate patient priorities and develop research agendas for medical products.^23^, ^27^ Finally, one group reported that PPI impacted their study by developing a series of initiatives designed to improve patient/researcher interactions such as patient‐focused webinars, panel discussions between researchers and patients and the preparation of patient‐focused sections in research publications.^28^

**Table 4:** Impact, evaluation and learning opportunities of included studies

References	Impact of PPI	Evaluation of PPI process	Potential learning opportunities for PPI in preclinical research
Birch et al.^22^	PPI contributors contributed to research activities including attending and contributing to scientific meetings, developing a glossary resource, contributing to a qualitative review paper, informed interview schedules and interpreting of qualitative data, assisted with the development of informational resources, evaluating a web‐based platform for communicating risk information of rheumatoid arthritis, developing patient questionnaires and informational resources, contributing to project website, developing lay summaries of research findings, designing posters for dissemination at conferences.	Qualitative and Quantitative Surveys. All PRPS reported a positive impact from their involvement. Mainly in terms of contributing their perspectives to researchers and their ability to communicate with the public.	Researchers and PRPs both reported they would have preferred more training in PPI. PPI contributors also expressed a desire for more ongoing feedback on the impact of their contribution to research activities.
Bridgelal Ram et al.^23^	Researchers and patients identified unmet needs and potential solutions for common EB issues during their PPI workshop. A design team used this data to develop products to meet this need. These products were then presented to the workshop participants for further discussion and refinement.	None reported.	PPI contributors living with serious conditions may become unwell, meaning researchers need to be flexible in organizing PPI sessions. The researchers also considered it important to involve clinicians and carers, who can contribute from their own experiences of working with serious conditions and may be required to administer any outputs from research.
Grier et al.^24^	Hosting a public meeting meant that potential PPI panel members had the opportunity to meet the research team and ask questions about what involvement constituted without committing. Some motivational factors for joining a PPI panel were identified relating to themes of concern with the impact of antimicrobial resistance in wider society, a sense of wanting to give something back and feeling as if they had something to offer.	None reported.	Researchers considered that having positive interactions with members of the research team may encourage people to become involved in PPI. PPI training for researchers was also seen as beneficial as lab‐based researchers generally have limited interaction with patients/service users.
Russel et al.^25^	Meeting attendees submitted a series of comments relating to the suitability of the research agenda presented by researchers for autism. The feedback demonstrated the diverse views of the autism community which was not represented initially by representatives of patient organizations. This is due to the researchers' initial PPI component only selecting patients from patient organizations that already supported their research agenda. This led to the classification of ‘selective PPI’ where only a sympathetic and/or limited is included in PPI.	None reported.	Representativeness is an important challenge for PPI. Preclinical researchers conducting PPI are at risk of only including patients who are sympathetic towards their viewpoints. Researchers should try to include a diverse set of views when conducting PPI.
Supple et al.^26^	PPI impacted the study in several ways. Patients collaborated with researchers on the submission of the funding application. PPI also altered the recruitment strategy for participants submitting biological samples for the preclinical project. PPI contributors enhanced the dissemination strategy by contributing to research papers from their patient perspectives. PPI contributors also helped draft lay summaries for each paper from the project and spoke at conferences about their experiences in the project.	None reported.	This study reports several learning outcomes for preclinical PPI: involve patients early where patient input is most impactful; involve patients deeply by having regular collaboration; provide patients with feedback on project progress; involve patients in the dissemination of research findings; allow patients to convey their own stories and experiences to help connect preclinical researchers with the needs of patients.
Elberse et al.^27^	Researchers and patients developed a report in collaboration with an independent advisory group that provides advice regarding public health policy to a national Minister for Health. In the report, patients articulated needs and outlined a research agenda for medical products regarding their conditions. This report was presented to the Minister for Health to inform a national research agenda for medical products.	None reported.	Researchers considered building relationships in the early stages of research important for collaborating with PPI contributors throughout the project. Researchers also found it beneficial to involve patients, carers and healthcare providers in PPI, due to their knowledge and experiences of particular conditions.
Baart and Abma^30^	Researchers and patients jointly submitted an action paper containing a series of recommendations for improving communication and interaction between researchers and patients, for example, redesigning the group website to make it more accessible for patients, using conferences to interact with patients and families, jointly hosting researcher/patient workshops and collaborating on publications.	None reported.	Researchers seeking to incorporate PPI should start by identifying areas of common interest between researchers and patients. Identifying common ground can serve as a basis for developing/strengthening relationships between groups of stakeholders.
Sohy et al.^28^	PPI had many impacts including hosting patient‐focused webinars for employees, panel discussions between researchers and patients, sending employees on visits to developing countries to see the impact of vaccines they produce and including patient‐focused sections in research publications.	Quantitative surveys reported that 72% of employees understood the purpose of the initiative and 65% reported improved patient interactions from the company with patients.	Preclinical researchers expressed a desire for increased interaction with patients in this study. Taking part in PPI may help preclinical researchers stay connected with the needs of patients who are served by their research.
Chalasani et al.^29^	Researchers strengthened their understanding of the disease burden for patients and their families as well as deepened their knowledge of the limitations and benefits of current treatment options. Meeting transcripts were posted online including a ‘Voice of the Patient’ summary report that captured PPI contributors' perspectives to provide further context from the panel meetings. Researchers used this input to provide patient context when advising drug development programmes and assessing products under review for market approval.	None reported.	Researchers published a summary of the meeting afterwards using PPI contributors' own words from the meeting transcript, webcast recording and comments submitted by PPI contributors attending the panel sessions.

Abbreviation: PPI, Patient and Public Involvement.

Only two studies reported formal evaluation of their PPI.^22^, ^28^ In one study, staff of a pharmaceutical company producing vaccines were surveyed (*n* = 743). The majority reported understanding the purpose of the PPI initiative (72%) and that it improved patient focus among researchers (65%), and 90% reported understanding the real‐world impact of their work.^28^ In another study, surveys designed in collaboration with PPI contributors were distributed to researchers (*n* = 15) and PPI contributors (*n* = 6) to collect feedback evaluating impact. All PPI contributors felt they had a positive impact on the research, and 73.4% of the researchers agreed.^22^ No other included study conducted a formal evaluation.

### 3.6 Learning opportunities for PPI in preclinical research

Included studies contained several key lessons that may be applied to PPI for preclinical research (Table 4). Researchers considered building relationships as a key enabler for preclinical PPI.^24^, ^27^, ^30^ Due to its long‐term nature, this was seen as particularly relevant for preclinical research.^27^ According to one study, researchers established relationships with PPI contributors by finding areas of similar scientific interest. This served as a basis for further discussions around involvement.^30^ Other considerations for relationship building were that it was most impactful at the early stages of research,^26^ and that a good relationship between researchers and PPI contributors improved the quality of PPI contributions to the research.^24^

Implementing PPI training was another learning point from the included studies. Two studies reported that training should be implemented for preclinical researchers conducting PPI.^22^, ^24^ One study, which evaluated their PPI, found that both researchers and PPI contributors would have preferred more training.^22^ Due to their limited level of interaction with patients generally, this was identified as particularly relevant for preclinical researchers.^22^, ^24^ Specifically, researchers in one study recommended that PPI training be integrated into basic training for research students.^22^

Providing PPI contributors with feedback on project progress was also important as described by three featured studies. According to the evaluation of one study, PPI contributors wanted feedback on their impact on research activities.^22^ In another study, researchers reported that providing regular project feedback improved PPI contributors' understanding of the research cycle.^26^ One study achieved this by publishing a summary of outputs in collaboration with PPI contributors to demonstrate the impact of PPI on their work.^29^

Researchers also reported that the selection of PPI contributors was an important consideration factor. Two studies described the benefits of involving clinicians, carers and family members in PPI, that is, those who can contribute different perspectives from their own experiences of working with serious conditions,^23^, ^27^ increasing the number of viewpoints provided. Similarly, representativeness was considered important for researchers conducting PPI,^25^ noting the potential to only involve a particular set of viewpoints. Only two studies included payment of PPI contributors.^22^, ^26^ and one study reported the authors' intention to do so in future studies.^24^

Finally, two studies discussed the importance of flexibility in organizing PPI.^23^, ^26^ Another consideration is the health of PPI contributors. In one study, researchers reported that PPI contributors living with epidermolysis bullosa frequently became unwell, requiring flexible planning to reschedule PPI workshops.^23^ Being flexible in organizing PPI sessions was important as researchers reported their sessions yielded rich data. Similarly, another study recommended that researchers should acknowledge the commitment of PPI contributors who may not be in good health, and facilitate involvement via phone and internet where possible.^26^

## 4 DISCUSSION

### 4.1 Summary of main findings and perspectives from PPI panel members

**Figure 2:**
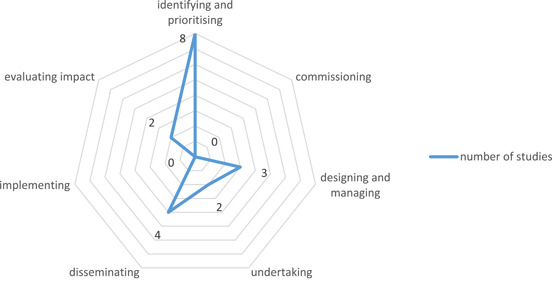
Frequency of stages of PPI implementation in included studies. PPI, Patient and Public Involvement.

This review aimed to map the breadth of literature for preclinical research studies conducting PPI. Only nine studies were included in the final review, suggesting that PPI is not regularly incorporated into preclinical research, as was originally hypothesized. In terms of aims and rationale, PPI was primarily included to ensure patients' voices informed the priorities of research, improving its relevance. To conduct PPI, researchers used a variety of approaches such as one‐off panel meetings or regular collaboration over the lifespan of a project as research partners. This may demonstrate a lack of standardization for preclinical PPI, or simply highlights the broad variety of approaches available, suggesting that PPI is applicable to the diverse nature of preclinical research. It is apparent that preclinical PPI is primarily focused on identifying research priorities (see Figure 2). No included studies conducted PPI at the commissioning or implementation stages of research.^1^ Unlike clinical health research where implementation may occur in policy or practice, for preclinical research, the implementation may involve progressing to further stages of research. Therefore, PPI may be considered less relevant in this context. Current PPI approaches in preclinical research are typically adopted from PPI in clinical research. While these serve as useful guides, clinical PPI approaches may not meet the needs of preclinical research. Therefore, there may be scope to develop PPI guidelines specific to preclinical research.

Benefits of conducting PPI included researchers improving their understanding of their area of research by building relationships with patients, thus seeing things from a new perspective. Challenges for preclinical PPI included ensuring PPI contributors' perspectives were representative of the patient population. Similarly, PPI contributors on our current PPI project noted that studies in the literature mostly involved patients and researchers, and indicated they would like to see further involvement of medical professionals in PPI, as they might be required to apply outcomes or administer any treatments that resulted from research outputs.

Examining impact, PPI primarily informed the priorities of research and influenced dissemination strategies of preclinical researchers. However, the stated positive impact must be balanced against the limited formal evaluation of PPI within these studies. One member of our PPI panel, providing their perspectives on the included studies, noted that preclinical researchers rarely reported their definition of PPI. Furthermore, they noted low adherence to PPI frameworks, such as those developed for the NHS,^31^ and PPI evaluation tools such as the Guidance for Reporting Involvement of Patients and the Public (GRIPP‐2) checklist^32^ thus limiting the impact of any findings.

Of the included studies, only two conducted a formal PPI evaluation.^22^, ^28^ It may be difficult to develop evidence‐informed guidance for preclinical PPI due to its lack of evaluation and limited evidence base. Furthermore, PPI is context specific and consists of various approaches, meaning its evaluation is difficult.^3^ This suggests that any guidance developed using the limited evidence base may help support meaningful preclinical PPI and help avoid tokenistic applications such as that critiqued by one included study.^25^

Two included studies reported using PPI to meet funding bodies' requirements, who often require evidence of PPI as part of funding applications.^29^, ^30^ This instrumental approach to PPI may result in tokenistic involvement. This risk was evident in one study where researchers reported initial hesitation toward PPI due to their belief that PPI contributors could not have a meaningful impact on a preclinical research study.^30^ However, engaging in PPI and talking with PPI contributors created a shift in researchers' position, and opened up further possibilities for collaboration. Therefore, funders necessitating PPI may be a positive development, as taking part in PPI may help preclinical researchers realize its benefits.

Finally, several learning points from included studies have applications to the PPI strategy of our own preclinical research project, such as taking time to build relationships with PPI contributors. While preclinical research may seem far removed from people living with a condition, they can still be involved in providing biological samples for analysis,^26^ or recruiting PPI panel members to oversee the work of a preclinical research project.^24^ A panel member from our project noted that PPI can help support the sustained engagement of PPI contributors by improving their experiences of being involved in research.

This study focused on PPI in preclinical research and identified a very small number of relevant studies when compared to reviews on PPI in clinical research.^4^ While the nature of the research being undertaken in clinical and preclinical settings is certainly different, some findings from this review resonated with those from previous clinical PPI studies. For example, despite the significantly larger literature base, reviews on PPI in clinical research also noted similar challenges such as recruiting a diverse range of representative PPI contributors and investing significant time and resources in PPI.^4^ However, while there has been sufficient research on PPI in clinical research to facilitate the conduct of systematic reviews and identification of benefits and impacts of PPI in that context, this review highlighted that this is not yet the case for preclinical PPI. Additionally, the majority of frameworks and tools are currently clinically orientated,^31^ which may limit their relevance in preclinical PPI and may need to be redesigned for use in preclinical research.

This co‐created scoping review provides a map of published preclinical research reporting primary empirical studies of preclinical PPI. When the team began designing and conducting this research, we aimed to collaboratively undertake a structured search of the literature to inform our own preclinical PPI strategy that would also serve as a resource for other preclinical researchers. In parallel, another review on patient engagement in preclinical laboratory research was conducted by researchers in Canada.^13^ This review reported related aims and objectives but was broader in scope with 30 papers identified. Search terms were broader and sources included reviews and opinion pieces, whereas this review focused on identifying and mapping primary empirical research and contextualizing the findings within our own PPI Advisory Panel and project. Three included studies were common to both reviews.^22^, ^25^, ^30^ While both reviews approach the topic from slightly different perspectives, both identified similar benefits and challenges associated with preclinical PPI, including the benefit of mutual learning between researchers and PPI contributors, and the challenges of representing diverse viewpoints held by people living with specific conditions. Both noted that preclinical PPI appeared to be primarily focused on the priority setting stage of research.

### 4.2 Implications

The few studies identified in this review indicate a lack of PPI use in preclinical research. The included studies appeared to report favourably on the potential for PPI in preclinical research, particularly in identifying research priorities. Growing the literature base of preclinical PPI would be important to identify broader trends. Furthermore, specialist training and budgeting may be needed to advance preclinical PPI. Importantly, researchers should also consider who to involve when conducting PPI. The majority of studies did not report paying PPI contributors for their time or reimbursing expenses. Allocating sufficient budgeting is important to ensure that the cost of attending meetings is not a barrier to engaging in preclinical PPI activities. Therefore, appropriate budgeting should be considered by preclinical researchers seeking to implement PPI in their work.^1^, ^33^ While patients and researchers are typically involved in PPI, medical professionals, carers and patients' family members may also be able to contribute from their unique and highly relevant perspectives. It may also be beneficial for preclinical researchers to focus on establishing good relationships with PPI contributors from the initial stages of research. Improving PPI contributors' experience with research may enable meaningful PPI and create further opportunities for PPI collaboration. Regular feedback may also enable the PPI process by improving PPI contributors' understanding of the research cycle. This may result in a sense of empowerment for PPI contributors from having tangible feedback on research. Finally, preclinical researchers should evaluate their PPI. This will contribute to the limited evidence base for preclinical PPI, and strengthen researchers' understanding of its impact on research.

### 4.3 Limitations

This review contains some limitations. First, while this study examined preclinical research studies which used PPI, two studies could be better described as reflections by preclinical researchers on their use of PPI.^24^, ^25^ Therefore, it was difficult to compare these studies with other studies containing dedicated PPI components. However, it was important to include these studies as they describe interesting cases of preclinical PPI, and provide valuable context for researchers learning about its application. Secondly, the search strategy may have missed some studies regarding PPI. The terminology surrounding PPI remains somewhat contested, with the ongoing debate on what constitutes PPI, patient engagement, CBPR, co‐production and a series of other terms describing involving patients in research.^34^ While the search strategy aimed to capture as much PPI literature as possible, and an updated search was conducted in August 2021 to capture updated search terms, some eligible studies may have been missed. Subsequent to our planning and completion of this review, specific JBI guidance on involving knowledge users has been published.^35^ We involved the PPI Advisory Panel members in most elements of the study, but not the screening and data extraction steps. It may be possible to further deepen involvement in future reviews by involving PPI Advisory Panel members in all stages as per the recent JBI guidance.

## 5 CONCLUSION

Preclinical researchers report limited use of PPI in their research and conduct a limited evaluation of their PPI. This limits its generalizability for other preclinical research studies. While the role of PPI may not be immediately apparent, preclinical researchers can use PPI to build positive relationships with PPI contributors, improving their knowledge of their research area, which will ultimately improve research outcomes for all stakeholders. Currently, there are a limited number of preclinical research studies incorporating PPI, suggesting an opportunity for the establishment of guidance on best practices. This is particularly relevant for preclinical researchers who have limited interaction with patients and may require guidance on the value and implementation of PPI.

## AUTHOR CONTRIBUTIONS

**Pádraig Carroll**: Conceptualization; data curation; formal analysis; investigation; methodology; project administration; visualization; writing – original draft. **Adrian Dervan**: Formal analysis; writing – review & editing. **Anthony Maher**: Conceptualization; formal analysis; writing – review & editing. **Ciarán McCarthy**: Conceptualization; formal analysis; writing – review & editing. **Ian Woods**: Formal analysis; writing – review & editing. **Rachel Kavanagh**: Formal analysis; writing – review & editing. **Cliff Beirne**: Formal analysis; writing – review & editing. **Geoff Harte**: Conceptualization; formal analysis; writing – review & editing. **Dónal O'Flynn**: Conceptualization; formal analysis; writing – review & editing. **Cian O'Connor**: Formal analysis; writing – review & editing. **Tara McGuire**: Formal analysis; writing – review & editing. **Liam M. Leahy**: Formal analysis; writing – review & editing. **Javier Gutierrez Gonzalez**: Formal analysis; writing – review & editing. **Martyna Stasiewicz**: Formal analysis; writing – review & editing. **Jack Maughan**: Formal analysis; writing – review & editing. **Pedro Jose Gouveia**: Formal analysis; writing – review & editing. **Paul J. Murphy**: Data curation; resources; writing – review & editing. **John Quinlan**: Formal analysis; writing – review & editing. **Sarah Casey**: Formal analysis; writing – review & editing. **Alice Holton**: Methodology; writing – review & editing. **Éimear Smith**: Formal analysis; supervision; writing – review & editing. **Frank Moriarty**: Formal analysis; methodology; supervision; writing – original draft; writing – review & editing. **Fergal J. O'Brien**: Conceptualization; formal analysis; funding acquisition; supervision; writing – review & editing. **Michelle Flood**: Conceptualization; data curation; formal analysis; funding acquisition; investigation; methodology; supervision; writing – original draft; writing – review & editing.

## CONFLICT OF INTEREST

The authors declare no conflict of interest.

## Supporting information

Supporting information.

## Data Availability

All data generated or analysed during the study are included in this published article (and its supplementary information files).
